# ABSCISIC ACID INSENSITIVE5 Interacts With RIBOSOMAL S6 KINASE2 to Mediate ABA Responses During Seedling Growth in *Arabidopsis*

**DOI:** 10.3389/fpls.2020.598654

**Published:** 2021-01-18

**Authors:** Linxuan Li, Tingting Zhu, Yun Song, Li Feng, Essam Ali Hassan Farag, Maozhi Ren

**Affiliations:** ^1^Institute of Urban Agriculture, Chinese Academy of Agricultural Sciences/Chengdu National Agricultural Science and Technology Center, Chengdu, China; ^2^Zhengzhou Research Base, State Key Laboratory of Cotton Biology, Zhengzhou University, Zhengzhou, China; ^3^Faculty of Agriculture, Al-Azhar University, Assiut, Egypt

**Keywords:** ABSCISIC ACID INSENSITIVE5, RIBOSOMAL S6 KINASE2, seedling growth, abscisic acid, AZD8055, *Arabidopsis*

## Abstract

ABSCISIC ACID INSENSITIVE5 (ABI5) is an important regulator of abscisic acid (ABA) signaling pathway involved in regulating seed germination and postgerminative growth in *Arabidopsis*, which integrates various phytohormone pathways to balance plant growth and stress responses. However, the transcriptional regulatory mechanisms underlying ABI5 and its interacting proteins remain largely unknown. Here, we found that inhibition of AtTOR could increase ABA content by up-regulating the expression levels of ABA biosynthesis-related genes, and thus activated the expression of ABA-responsive genes. Pharmacological assay showed that *abi5-1* mutant was insensitive to TOR inhibitor AZD8055, whereas *AtABI5* overexpression lines were hypersensitive to AZD8055 in *Arabidopsis*. Biochemical interaction assays demonstrated that ABI5 physically interacted with the RIBOSOMAL S6 KINASE2 (S6K2) protein in plant cell. S6K2 positively regulated ABA responses during seedling growth and upregulated ABA-responsive genes expression. Furthermore, genetic and physiological analysis indicated that *AtS6K2* overexpression lines enhanced resistance to drought treatment while *AtS6K2* interference lines were sensitive to drought. These results indicated that AtABI5 interacted with AtS6K2 to positively modulate ABA responses during seedling growth and shed light on a underlying mechanism of the crosstalk between TOR and ABA signaling pathways in modulating seedling growth in *Arabidopsis*.

## Introduction

Plants have evolved a set of precise regulatory mechanisms to adapt to environmental changes and resist to biotic or abiotic stresses. Abscisic acid (ABA) is a key plant hormone for plant resistance to abiotic stress (Vishwakarma et al., 2017). ABA signaling pathway plays a critical role in regulating various developmental processes and resistance to abiotic stress in plants (Cutler et al., 2010). PYR/PYL/RCAR family members are found as ABA receptors and inhibit the activity of type 2C protein phosphatases (PP2Cs) to control ABA signaling (Nishimura et al., 2010; Pizzio et al., 2013). ABA insensitive 1 (ABI1) and ABI2, which are prototypical members of PP2Cs, are early negative regulators in ABA signal transduction and inhibit the activity of SNF1-related protein kinase 2 (SnRK2) (Bertauche et al., 1996). *snrk2.2/2.3/2.6* triple mutant was shown to cause a strong ABA insensitive phenotype in seed germination and seedling growth, and ABA-activated protein kinases activity and ABA-induced genes expression are blocked in the triple mutant (Fujii and Zhu, 2009; Nakashima et al., 2009). SnRK2s protein kinases are required for ABA response and capable of phosphorylating downstream transcription factors such as ABA insensitive 5 (ABI5) and ABRE-binding factors (ABFs) to regulate the expression of ABA-responsive genes (Fujii et al., 2007).

The target of rapamycin (TOR) is an evolutionally conserved protein kinase that regulates cell metabolism, nutrition, growth, and development in response to environmental cues in various eukaryotic species (Wullschleger et al., 2006; Ren et al., 2012; Yuan et al., 2013). TOR and other proteins make up TOR complex 1 (TORC1) and TORC2 (Loewith et al., 2002). TORC1 is sensitive to rapamycin and contains TOR, regulatory-associated protein of TOR (RAPTOR) and lethal with SEC13 protein 8 (LST8). The core of rapamycin-insensitive TORC2 complex includes TOR, LST8, rapamycin-insensitive companion of TOR (RICTOR) and stress activated map kinase-interacting protein 1 (SIN1) (De Virgilio and Loewith, 2006; Wullschleger et al., 2006). In *Arabidopsis*, only one single *TOR* (*AtTOR*) gene was identified (Menand et al., 2002). The AtTOR protein shares high similarities with yeast and human TOR proteins. The homologs of TORC1 components and downstream effectors have been identified in *Arabidopsis* and other photosynthetic plants (Creff et al., 2010; Ahn et al., 2011; Ren et al., 2012). Ribosomal S6 kinases (S6Ks) are well known as the major substrates of TOR kinase and mediate TORC1 signaling for ribosome biogenesis, protein synthesis and cell proliferation in eukaryotes (Magnuson et al., 2012). Both mammals and plants have two types of S6Ks, namely S6K1 and S6K2 (Henriques et al., 2010; Magnuson et al., 2012). Although the biological functions of S6K1 and S6K2 are partially redundant, recent research showed that S6K2 has biological functions distinct from those of S6K1 in mammals and plants (Pardo and Seckl, 2013; Xiong et al., 2017).

Rapamycin (RAP), produced by *Streptomyces hygroscopicus*, is a macrocyclic lactone metabolite and is regarded as the first generation inhibitor of TOR (Vezina et al., 1975). RAP forms a ternary complex with the FRB domain of TOR and the FK506 binding protein of 12kDa (FKBP12), eventually inhibits the TORC1 activity in yeast and animals (Loewith et al., 2002). However, because of the structure alteration of FKBP12, RAP fails to bind FKBP12, resulting in RAP-insensitive phenotype in most plants (Xu et al., 1998; Sormani et al., 2007). The RAP-insensitivity makes it difficult to identify TOR downstream effectors in photosynthetic plants (Xu et al., 1998). To gain further insight into TOR signaling by RAP, Ren et al. (2012) generated a yeast *FKBP12*-based *Arabidopsis* transgenic line (BP12-2), which is hypersensitive to RAP. Meanwhile, recent study showed that *Arabidopsis* FKBP12 could form a tertiary complex with rapamycin and the FRB domain of AtTOR at higher rapamycin concentrations. The variable endogenous FKP12 protein levels may offer a molecular explanation for observations on inconsistent rapamycin resistance in plants and animals (Xiong and Sheen, 2012). Besides, the active-site TOR inhibitors (asTORis) including AZD8055 (AZD), KU0063794, Torin1, and Torin2 are also well developed to study TOR signaling pathway (Garcia-Martinez et al., 2009; Chresta et al., 2010; Liu et al., 2011). asTORis bind to the kinase domain of TOR to inhibit both the activity of TORC1 and TORC2 in mammalian (Garcia-Martinez et al., 2009; Liu et al., 2011). Recent study showed that asTORis inhibit whole-plant growth in a dosage dependent manner in various plants (Montane and Menand, 2013). Especially, AZD is a potent and highly selective inhibitor of TOR kinase in *Arabidopsis* (Montane and Menand, 2013; Li et al., 2015). The application of asTORis in plants provides an efficient means to study plant TOR signaling pathway.

Target of rapamycin is a key regulator of plant growth and development. Down-regulation of *AtTOR* expression leads to a post-germinative halt in growth and development, which phenocopies the action of the plant hormone ABA (Deprost et al., 2007). Expression profiling in *Arabidopsis* revealed that inhibition of TOR results in the differential expression of ABA signal transduction related genes (Dong et al., 2015). Recent study revealed that the TOR kinase phosphorylates ABA receptors to inhibit stress responses under normal conditions. Under stress conditions, ABA-activated SnRK2s phosphorylates RAPTOR, a regulatory component of TORC1, to prevent plant growth and activate the expression of ABA-responsive genes by inhibiting TOR activity (Wang et al., 2018). Besides, ABI4, a key transcription factor of ABA signaling pathway, is identified as a downstream effector of TOR signaling by a chemical genetics approach (Li et al., 2015). TAP42 interacting protein of 41 kDa (TIP41), the downstream component of the TOR signaling pathway, is also involved in the regulation of the ABA signaling pathway. The *tip41* mutant was highly sensitive to exogenous ABA and NaCl during seed germination and seedling growth. Overexpression *TIP41* plants were able to tolerate exogenous ABA and NaCl, indicating that TIP41 can coordinate plant growth and stress responses in *Arabidopsis* (Punzo et al., 2018). These researches provide essential clues for the crosstalk between TOR and ABA signaling pathways. However, whether the key node genes in ABA signal transduction are involved in the crosstalk between TOR and ABA signaling pathways still need further study. Therefore, it is of great interest to understand the complex regulatory mechanisms between TOR and ABA signals.

In this study, we found that TOR inhibitors and ABA synergistically inhibited the seeding growth of *Arabidopsis*. Inhibition of AtTOR increased ABA content by up-regulating the expression levels of ABA biosynthesis-related genes. Pharmacological assay showed that *abi5-1* mutant was insensitive to TOR inhibitor AZD, whereas *AtABI5* overexpression (OE) lines were hypersensitive to AZD in *Arabidopsis*. Yeast two-hybrid and bimolecular fluorescence complementation (BiFC) assays demonstrated that ABI5 physically interacted with the S6K2 protein in plant cell. *AtS6K2* OE lines displayed the ABA hypersensitive phenotypes at seedling stage as well as a upregulation of ABA-responsive genes. Furthermore, *AtS6K2* OE lines showed enhanced resistance to drought treatment while *AtS6K2* interference line was sensitive to drought. Collectively, these results indicated that AtABI5 interacted with AtS6K2 to positively modulate ABA responses during seedling growth in *Arabidopsis*.

## Materials and Methods

### Plant Materials and Growth Conditions

*Arabidopsis* seeds were surface sterilized by using liquid methods. The seeds first treated with 70% ethanol for 2 min and the supernatant was discarded; then, with 10% sodium hypochlorite containing 0.3% Tween-20 for 5 min, and the supernatant was discarded, followed by rinses with sterile water four times, centrifugation for 2 min at 4,000 *g* each time, and the supernatant was discarded. Finally, the seeds were suspended in 0.15% sterile agarose and kept at 4°C for 2 days. Sterilized seeds were plated on plates and grown in a growth chamber at 22°C under 16 h 60–80 μE m^–2^ s^–1^ continuous light and 8 h darkness. The *abi5-1* mutant line in Ws background was obtained from Dr. Zhizhong Gong. The transgenic *Arabidopsis* BP12-2 was generated in the previous study (Ren et al., 2012).

### Overexpression Constructs and Transformation of *Arabidopsis* Plants

Total RNA of the Col-0 (WT) seedlings was isolated using the RNAprep Pure Plant Kit (TIANGEN, Beijing, China). Full-length CDS were amplified by PCR using the TransStar Taq Polymerase Mix kit (TRANSGEN, Beijing, China) following the manufacturer’s instructions. The corresponding restriction enzyme sites were introduced into 5′ and 3′ end of the respective primer. The recombinant OE constructs *P35S*::*AtABI5*-HA, *P35S*::*AtABI5*-*GUS*, *P35S*::*AtS6K2*-HA, and RNAi construct *P35S*::*AtS6K2*-RI (interference with *AtS6K2*) driven by the CaMV 35S promoter were generated as previously described (Ren et al., 2011).

These constructs were introduced into *Agrobacterium tumefaciens* strain GV3101 and used in the transformation of WT Col-0 plants. Transgenic *Arabidopsis* lines were generated by *Agrobacterium* mediated transformation using the floral dip method. T1 transgenic seeds were selected on half-strength Murashige and Skoog medium (1/2 MS) medium containing 50 mg/L kanamycin, and T3 homozygous progeny were used for further study. At least two independent lines of each transgenic material were used in this study.

### Measurement of Seed Germination and Cotyledons Greening Ratio

The germination and cotyledons greening ratio of indicated seeds were performed as described previously (Kai et al., 2013). Seeds were grown on 1/2 MS supplemented with 0.5 μM ABA, 0.5 μM AZD, and 0.35 μM RAP, respectively. Germination was determined based on the appearance of the embryonic axis. Cotyledon greening was based on the observation of green cotyledons in a seedling. Three independent experiments were performed and similar results were observed. ABA, AZD, and RAP were dissolved in dimethyl sulfoxide (DMSO), and DMSO was used as a solvent control.

### Combination Index Value Measurement

Combination index (CI) values were used to evaluate the interaction between RAP/AZD and ABA. The degree of reagents interaction is based on synergistic effect (CI < 1), additive effect (CI = 1), or antagonism (CI > 1) (Chou, 2006). BP12-2 and WT seeds were sown on plates containing DMSO, different concentrations of ABA, RAP, AZD and pairwise combination for 10 days, and fresh weight was measured for CI value assessment. Experiments were repeated three times. The values of affected fraction (Fa) were calculated according to the CompuSyn software program. The value of Fa indicated plant growth inhibition affected by the reagent and was calculated according to the software instructions as follows: (1-T/C) ×100%. C: fresh weight of control plants, T: fresh weight of drug-treated plants.

### Quantitative Real-Time PCR

Total RNA of WT seedlings treated for 48 h on mediums containing DMSO, ABA (50 μM), AZD (2 μM), and ABA (50 μM) + AZD (2 μM) was isolated using the RNAprep Pure Plant Kit (TIANGEN, Beijing, China). Total RNA was treated with RNase-free DNase. PrimeScript^®^ RT reagent kit (Takara, Dalian, China) was used for reverse transcription, following the manufacturer’s instructions. Relative transcript levels were assayed by two-step real-time PCR analysis using the CFX96 real-time PCR system (Bio-Rad, United States). Real-time primers were designed by Primer Premier 5.0 and the details are presented in Supplementary Table 1. *AtACTIN2* was used as an internal control. Reactions were performed in a final volume of 20 μL containing 10 μL of 2 × SYBR Premix Ex *Taq* II (Takara, Dalian, China). Genes relative quantification analyses were performed with the formula: 2^–ΔΔ*Ct*^. The data represented the mean ± SD of three independent experiments. Each data point was determined in triplicate in each of the three biological replicates and expressed as the mean ± SD.

### GUS Staining and Quantitative Determination of GUS Activity

Transgenic plants staining for GUS activity using X-Gluc were performed as previously described (Menand et al., 2002). The transgenic plants were incubated with GUS staining solution for 5 h at 37°C, and a 70% ethanol wash was performed to remove chlorophyll from the leaves. Images were captured using a stereomicroscope (OLYMPUS MVX10, Japan). Each treatment containing three biological replicates. Quantitative GUS assay was performed using the MarkerGene^TM^β-glucuronidase (GUS) reporter gene activity detection kit (Marker Gene Technologies, Inc., Eugene, OR, United States). Total proteins in extracts of the transgenic plants were quantified using the Bradford assay.

### ABA Content Measurement

Seven-day-old *Arabidopsis* seedlings were transferred to 1/2 MS medium containing DMSO, AZD (2 μM), RAP (5 μM), and AZD (2 μM) + RAP (5 μM) for 48 h. Each treated *Arabidopsis* seedlings were harvested for measurement of ABA content. ABA was extracted following the exact extraction procedure. ABA extraction was quantified by LC-MS methods as previously reported (Kai et al., 2013). Three biological replications were performed.

### Yeast Two-Hybrid Assay

In order to construct plasmids for yeast two-hybrid analyses, the coding sequences of *AtABI5*, *AtRAPTOR1B*, *AtS6K1*, *AtS6K2*, and *AtBIN2* genes were amplified from the cDNA of *Arabidopsis*. The *AtABI5*, *AtABI5-N*, and *AtABI5-C* were inserted into the vector pGADT7. *AtRAPTOR1A*, *AtRAPTOR1B*, *AtS6K1*, *AtS6K2*, and *AtBIN2* genes were cloned into vector pGBKT7. The pairs of yeast two-hybrid plasmids were co-transformed into *S*accharomyces *cerevisiae* strain Y2H Gold following the PEG/LiAc transformation protocol (Clontech, Cat. no. 630489). In addition, the plasmids pGBKT7-53 and pGADT7 served as a positive control. The pGBKT7-Lam and pGADT7 plasmids were used as negative control. Transformants were grown at 28°C for 5 days on synthetic medium lacking Leu and Trp, then yeast colonies were transferred to synthetic medium stripped of His, Leu, Ade, and Trp containing 40 μg/mL X-a-Gal and 200 ng/mL Aureobasidin A as described by the manual (Clontech, Cat. no. 630489). Three independent experiments were performed.

### Bimolecular Fluorescence Complementation Assay

The *AtABI5* full-length CDS sequence was cloned into the BiFC vector pAB855-cYFP vector, and the full-length CDS sequence of *AtS6K2* was cloned into the BiFC vector pAB862-nYFP vector. The constructed BiFC vectors *AtABI5*-cYFP and *AtS6K2*-nYFP were transferred into the *Agrobacterium* GV3101 strain. The constructs were transiently coexpressed in tobacco leaves (*Nicotiana benthamiana*) performed as described previously (Hu and Yu, 2014). The images were captured using a confocal laser-scanning microscope (Leica TCSSP8, Germany).

## Results

### TOR Inhibitors and ABA Synergistically Inhibit the Growth of *Arabidopsis* Seedlings

**FIGURE 1:**
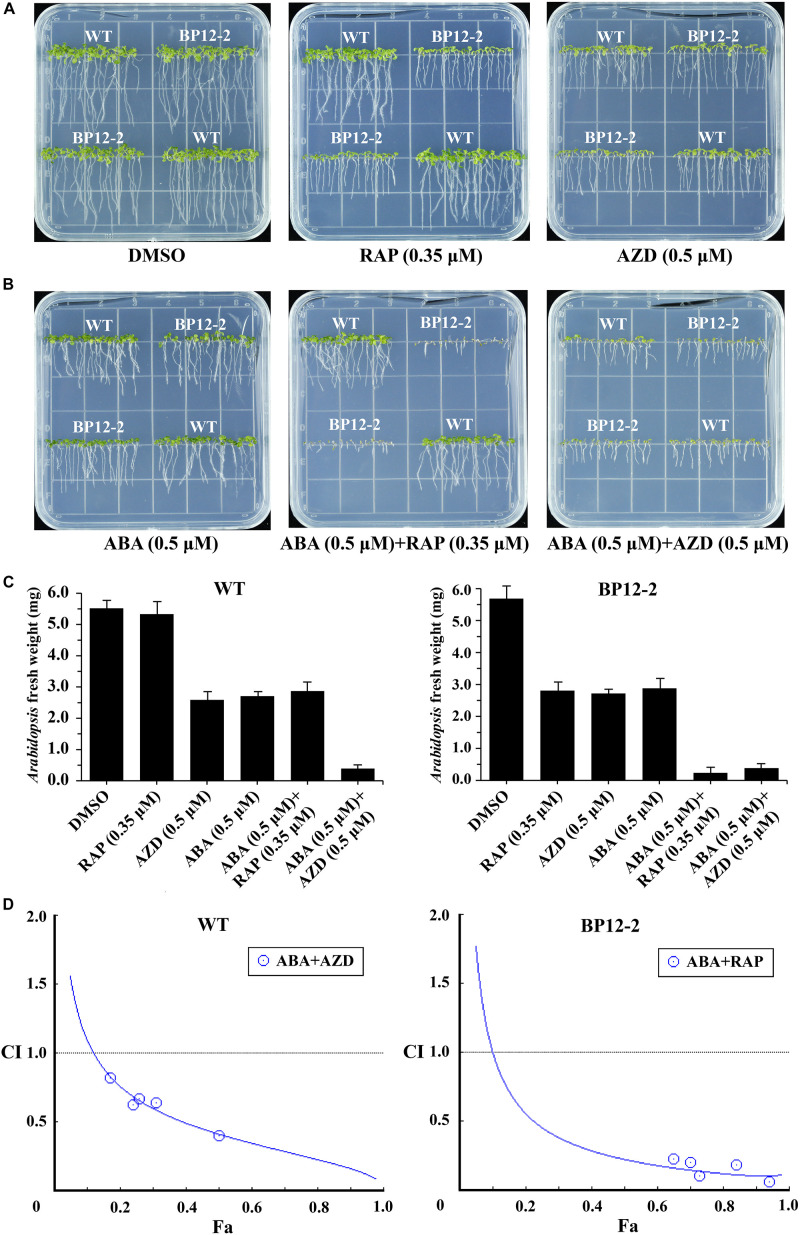
ABA and TOR inhibitors inhibit seedling growth and development in *Arabidopsis*. **(A)** Phenotypes of 10-day-old WT (Col-0) and BP12-2 seeds sown on plates containing DMSO, RAP (0.35 μM), and AZD (0.5 μM). **(B)** Phenotypes of 10-day-old WT and BP12-2 seeds sown on plates containing ABA (0.5 μM) and the combination of ABA (0.5 μM) + RAP (0.35 μM), and ABA (0.5 μM) + AZD (0.5 μM). **(C)** Fresh weight of BP12-2 and WT seeds sown on various plates for 10 days. Each graph represents the average of 30 seedlings. Error bars indicate means ±SD of three biological replicates. **(D)** ABA and TOR inhibitors synergistically inhibit plant growth in *Arabidopsis*. BP12-2 and WT seeds were sown on plates containing DMSO, different concentrations of ABA, RAP, AZD and pairwise combination for 10 days, and fresh weight of seedlings was measured for CI value assessment. The Fa-CI curve shows synergistic effects (CI < 1) between ABA + RAP and ABA + AZD in BP12-2 and WT seedlings, respectively.

Target of rapamycin and ABA signaling pathways play important roles in integrating stress responses with plant growth. Recent studies have found the interactions between the TOR and ABA pathways (Punzo et al., 2018; Wang et al., 2018). To further dissect the interactions between the TOR and ABA signaling pathways, we used the TOR kinase inhibitors rapamycin (RAP) and AZD and plant hormone ABA to treat *Arabidopsis* seeds. RAP-sensitive transgenic *Arabidopsis* line (BP12-2) expressing yeast FK506 binding protein12 (ScFKBP12) was recruited to test the interactions between the RAP and ABA. RAP had no significant inhibitory effect on the Col-0 wild-type (WT), but had a significant inhibitory effect on BP12-2 line, resulting in slower leaf growth, shortened root length and reduced root hair (Figure 1A), which is consistent with the findings of previous reports (Ren et al., 2012). AZD and ABA inhibited seedling cotyledons growth, chlorophyll accumulation and root elongation in a dose-dependent manner in both BP12-2 and WT plants. Besides, we found that TOR kinase inhibitors RAP and AZD had no effect on seed germination, but BP12-2 and WT plants showed hypersensitivity to ABA since 0.5 μM decreases germination by 70% (Supplementary Figure 1). According to the results of *Arabidopsis* fresh weight, the 50% growth inhibitory dose (GI50) of RAP, AZD and ABA approximately were 0.35, 0.5, and 0.5 μM, respectively (Figures 1A–C). Furthermore, the GI50 concentration combination of RAP and ABA showed that cotyledons could not expand and turn green, hypocotyls and roots could not elongate, and the seedlings almost stopped growing. These results implied that TOR and ABA signals may be involved in the regulation of plant growth and stress responses together in *Arabidopsis*.

To further test whether TOR inhibitors and ABA synergistically inhibit seedling growth, we used a CI to calculate the interaction between TOR inhibitors and ABA in *Arabidopsis*. The combination treatment of ABA + RAP generated the strong synergistic effects (CI < 0.5) in BP12-2 plants. Meanwhile, the combination treatment of ABA + AZD also generated the synergistic effects (CI < 1) in WT plants (Figure 1D). Furthermore, the pairwise combination of ABA + RAP or ABA + AZD had enhanced inhibition for plant growth compared with ABA or TOR inhibitors alone treatment. The GI50 values of each drug used in combination were significantly lower than that of each drug used alone (Supplementary Table 2). These results showed that ABA and TOR inhibitors may synergistically inhibit the growth of *Arabidopsis* seedlings.

### TOR Is Involved in the Regulation of ABA Biosynthesis and ABA-Induced Genes Expression

**FIGURE 2:**
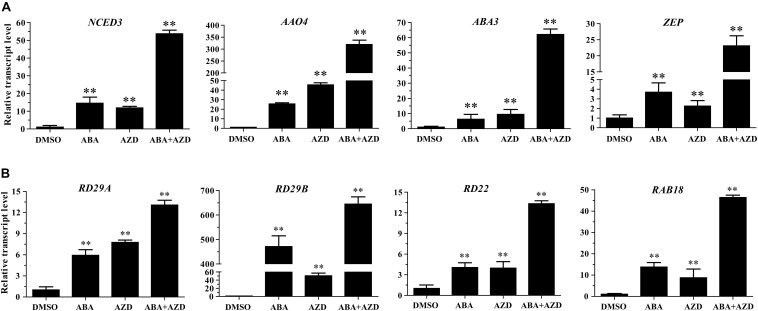
Relative transcription levels of ABA biosynthesis genes and ABA response genes in *Arabidopsis* seedlings. **(A)** qRT-PCR analysis of *NCED3*, *AAO4*, *ABA3*, and *ZEP* transcript levels in 7-day-old WT seedlings treated with DMSO, ABA (50 μM), AZD (2 μM), and ABA (50 μM) + AZD (2 μM) for 48 h. The data represents the mean ± SD of *n* = 3 independent experiments. Asterisks denote Student’s *t*-test significant difference compared with DMSO (^∗∗^*P* < 0.01). **(B)** qRT-PCR analysis of *RD29A*, *RD29B*, *RD22*, and *RAB18* transcript levels in 7-day-old WT seedlings treated with DMSO, ABA (50 μM), AZD (2 μM), and ABA (50 μM) + AZD (2 μM) for 48 h. The data represents the mean ± SD of *n* = 3 independent experiments. Asterisks denote Student’s *t*-test significant difference compared with DMSO (^∗∗^*P* < 0.01).

**FIGURE 3:**
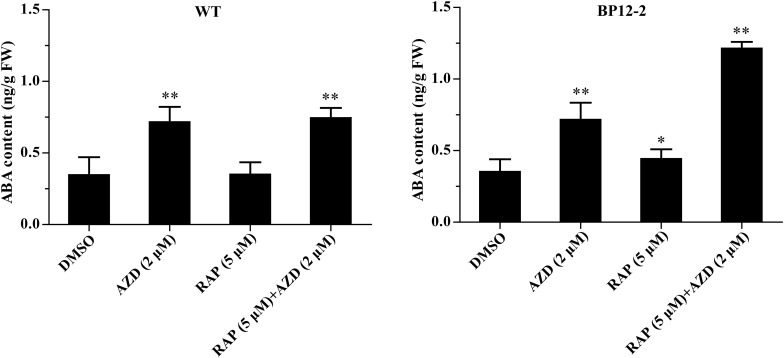
ABA content in WT and BP12-2 seedlings. WT and BP12-2 seedlings were grown for 7 days on 1/2 MS medium and then transferred to plates containing DMSO, AZD (2 μM), RAP (5 μM), and RAP (5 μM) + AZD (2 μM) for 2 days. The data represents the mean ± SD of three biological experiments. Asterisks denote Student’s *t*-test significant difference compared with DMSO (^∗^*P* < 0.05; ^∗∗^*P* < 0.01). FW, fresh weight.

In the previous experiments, we found that *Arabidopsis* seedlings are hypersensitive to ABA under TOR inhibition. To investigate whether TOR inhibition induced ABA accumulation, we analyzed the transcription levels of ABA biosynthesis-related genes. *9-cis-epoxycarotenoid dioxygenase 3* (*NCED3*) is a key rate-limiting enzyme in ABA biosynthesis. *AtNCED3* is induced by drought stress and controls the content of endogenous ABA under drought conditions in *Arabidopsis* (Iuchi et al., 2001). The transcription level of *AtNCED3* was significantly increased under TOR inhibition by AZD. The transcription levels of other ABA biosynthesis-related genes including *abscisic aldehyde oxidase 4* (*AAO4*), *abscisic acid deficient 3* (*ABA3*) and *zeaxanthin epoxidase* (*ZEP*) were also significantly up-regulated under TOR inhibition. In addition, the transcription levels of these genes were significantly induced in seedlings treated with ABA or ABA + AZD (Figure 2A). To further verify whether TOR inhibition can induce ABA biosynthesis, we used LC-MS to detect ABA content in WT and BP12-2 seedlings treated with TOR inhibitors. There was no significant difference in ABA content between RAP- and DMSO-treated WT plants, and the ABA content had no obvious difference in DMSO control group of BP12-2 and WT plants. However, the endogenous ABA content was significantly increased in BP12-2 seedlings treated with RAP or AZD (Figure 3). Furthermore, the ABA content of RAP + AZD combination was significantly higher than that of RAP or AZD alone in BP12-2 seedlings (Figure 3). These results indicated that inhibition of AtTOR could increase ABA content by up-regulating the expression levels of ABA biosynthesis-related genes.

Previous studies have shown that ABA can bind to ABA receptor PYLs to activate SnRK2s protein kinase in plants. Activated SnRK2s promote the expression of ABA-responsive genes by phosphorylating transcription factors such as ABFs and ABI5 (Fujita et al., 2009; Yue et al., 2009; Nishimura et al., 2010). To verify whether the TOR inhibition can activate the expression of ABA-responsive genes, we analyzed the expression levels of ABA-responsive genes in AZD-treated WT *Arabidopsis* seedlings. Transcription levels of ABA-responsive genes such as *responsive to desiccation 29A* (*RD29A*), *responsive to desiccation 29B* (*RD29B*), *responsive to dehydration 22* (*RD22*), and *RAB GTPase homolog B18* (*RAB18*) were significantly up-regulated in ABA and AZD-treated WT *Arabidopsis* seedlings (Figure 2B). Among these genes, the up-regulated level of *RD29B* gene was highest, and the up-regulation ratio of *RD22* gene was the lowest in ABA and AZD treatment. The expression levels of *RD29A*, *RD29B*, *RD22*, and *RAB18* genes in ABA + AZD combination treatment were significantly higher than that of ABA or AZD alone. These results showed that AtTOR inhibition activates the expression of ABA-responsive genes by inducing ABA biosynthesis in *Arabidopsis*.

### ABI5 Mediates the Transduction of TOR Signal in Plant Growth

**FIGURE 4:**
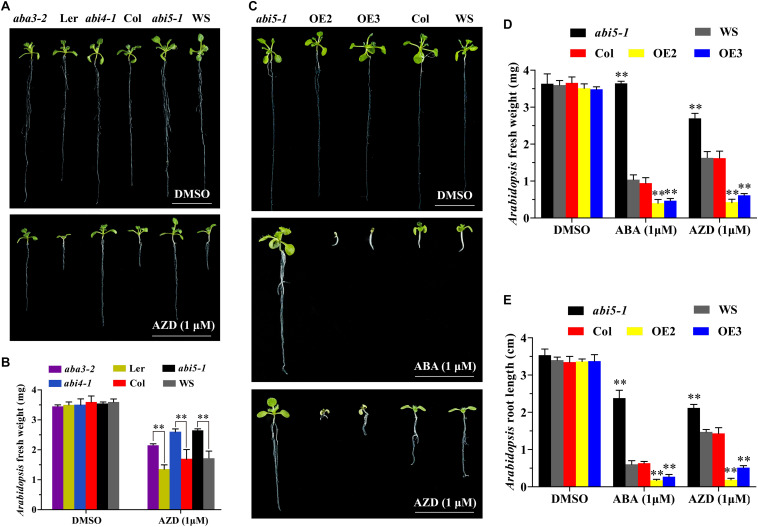
Effect of AZD on ABA biosynthesis and transduction deficient mutants. **(A)** Phenotypes of *aba3-2*, *abi4-1*, and *abi5-1* mutant seeds sown on plates containing DMSO and AZD (1 μM) for 10 days. Bar, 1 cm. **(B)** Fresh weight of *aba3-2*, *abi4-1*, and *abi5-1* mutant seeds sown on plates containing DMSO and AZD (1 μM) for 10 days. Each graph represents the average of 10 seedlings. Error bars indicate ±SD of three biological experiments. Asterisks denote Student’s *t*-test significant difference (^∗∗^*P* < 0.01). **(C)** Phenotypes of *abi5-1*, *ABI5-HA* overexpression lines (OE) and WT seeds sown on plates containing DMSO, ABA (1 μM), and AZD (1 μM) for 10 days. Bar, 1 cm. **(D,E)** Fresh weight and root length of *abi5-1*, *ABI5-HA* overexpression lines (OE) and WT seeds sown on plates containing DMSO, ABA (1 μM), and AZD (1 μM) for 10 days. Each graph represents the average of 10 seedlings. Error bars indicate ±SD of three biological experiments. Asterisks denote Student’s *t*-test significant difference compared with WT (^∗∗^*P* < 0.01).

To further investigate the interaction between TOR and ABA signals, we observed the growth of the ABA biosynthesis and signal transduction related mutants including *aba2-3*, *aba3-2*, *abi1-1*, *abi2-1*, *pyl4-1, abi4-1*, and *abi5-1* under TOR inhibition. The results showed that AZD had no effect on the growth of *aba2-3*, *abi1-1*, *abi2-1*, and *pyl4-1* mutants compared with WT *Arabidopsis* plants. However, ABA biosynthesis mutant *aba3-2* was insensitive to AZD compared with L*er*, which reflected in longer roots, larger leaves and heavier fresh weight (Figures 4A,B). Moreover, consistent with the findings of previous reports (Li et al., 2015), the transcription factor *abi4-1* mutant was insensitive to AZD compared with Col plants. Interestingly, the other transcription factor of ABA signal *abi5-1* mutant was also insensitive to AZD compared with WS plants (Figures 4A,B). It was worth noting that AZD inhibited the growth of *aba3-2*, *abi4-1*, and *abi5-1* mutants compared with DMSO, especially root growth (Figures 4A,B). To further decipher the functions of ABI5 in *Arabidopsis*, we generated 35S promoter driven-*ABI5* OE plants by introducing *P35S*::*ABI5*-HA construct into Col-0 *Arabidopsis*. Contrary to *abi5-1* mutant, *ABI5-HA* OE lines showed sensitive to ABA and AZD, resulting in shorter roots and shoots, bleached cotyledons and seedling growth retardation (Figures 4C–E and Supplementary Figure 2).

**FIGURE 5:**
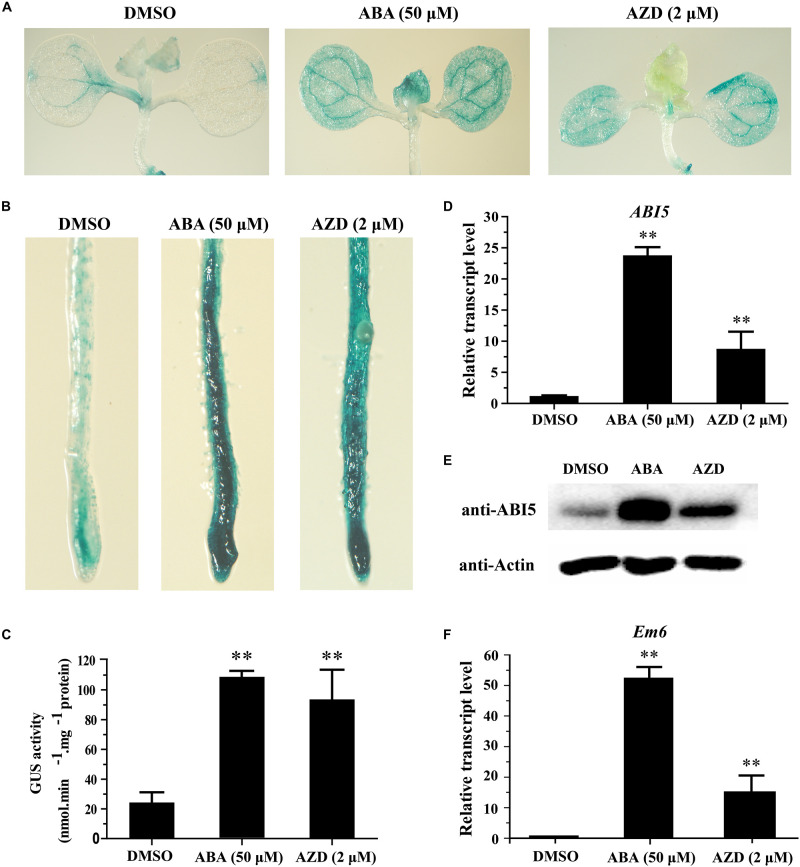
Excessive accumulation of ABI5 protein in plants with ABA and AZD treatment. **(A)** GUS staining of leaves of 7-day-old *ABI5-GUS* OE5 plants treated with DMSO, ABA (50 μM), and AZD (2 μM) for 48 h. **(B)** GUS staining of roots of 7-day-old *ABI5-GUS* OE5 plants treated with DMSO, ABA (50 μM), and AZD (2 μM) for 48 h. **(C)** GUS activity of *ABI5-GUS* OE5 seedlings, plants were treated as described in **(A)**. Asterisks denote Student’s *t*-test significant difference compared with DMSO (^∗∗^*P* < 0.01). **(D)** Relative transcript level of *ABI5* gene in 7-day-old Col plants treated with DMSO, ABA (50 μM) and AZD (2 μM) for 48 h. **(E)** Western blot of 7-day-old Col plants treated with DMSO, ABA (50 μM), and AZD (2 μM) for 48 h. **(F)** Relative transcript level of *Em6* gene in 7-day-old Col plants treated with DMSO, ABA (50 μM), and AZD (2 μM) for 48 h. Error bars indicate ±SD of three biological experiments. Asterisks denote Student’s *t*-test significant difference compared with DMSO (^∗∗^*P* < 0.01).

ABSCISIC ACID INSENSITIVE5 is an important transcription factor in seed germination and seedling growth, the protein level of ABI5 is finely regulated by proteasome (Stone et al., 2006; Yu et al., 2015). Excessive accumulation of ABI5 protein can hinder the growth of plant seedlings. To test whether TOR signal regulates the growth and development of *Arabidopsis* by regulating the stability of ABI5 protein, we fused *ABI5* with *GUS* and generated *P35S*::*ABI5*-*GUS* transgenic *Arabidopsis*. High level of GUS activity was observed in leaves and root tips of *P35S*::*ABI5*-*GUS* transgenic plants treated with ABA and AZD (Figures 5A,B and Supplementary Figure 3). The quantitative results suggested that the GUS signal was significantly increased in *P35S*::*ABI5*-*GUS* transgenic plants treated with ABA and AZD, whereas the GUS activity of AZD treatment was significantly lower than that of ABA treatment (Figure 5C). We analyzed the expression of *ABI5* gene in transcription and protein levels. The results showed that the transcript level of *ABI5* was significantly up-regulated in ABA and AZD-treated plants (Figure 5D). The western blot results showed that ABA and AZD induced excessive accumulation of ABI5 protein in *Arabidopsis* (Figure 5E). We further examined the expression level of *early methionine-labelled 6* (*Em6*), which is a known ABI5 target gene (Carles et al., 2002). Consistently, the expression level of *Em6* was strongly increased in ABA and AZD-treated plants (Figure 5F). Together, these results demonstrated that AtTOR inhibition promotes the accumulation of ABI5 protein, which induced the expression of ABI5 target genes.

### AtABI5 Interacts With AtS6K2

**FIGURE 6:**
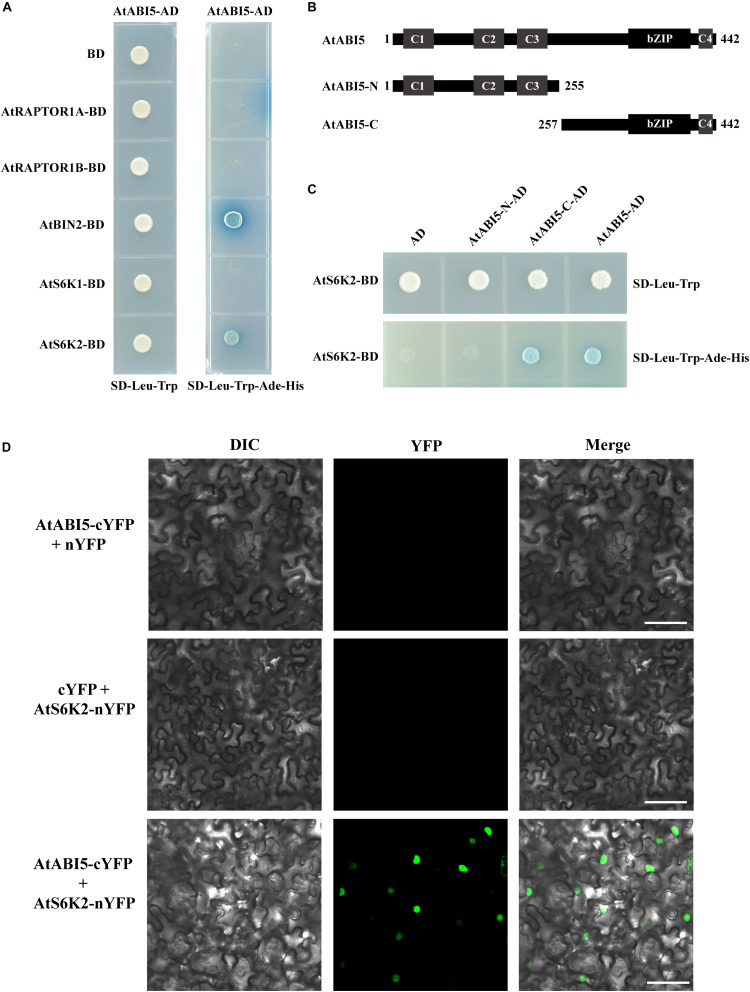
AtABI5 interacts with AtS6K2. **(A)** AtABI5 interacts with AtS6K2 in Y2H assay. *AtABI5* was inserted into pGADT7 (AD) vector, and *AtRAPTOR1A*, *AtRAPTOR1B*, *AtBIN2*, *AtS6K1*, and *AtA6K2* genes of TOR signaling pathway were inserted into pGBKT7 (BD). Interaction was indicated by the ability of yeast grown on dropout medium lacking Leu, Trp, Ade, and His. **(B,C)** AtS6K2 interacts with N-terminus 255 amino acid truncated AtABI5 (AtABI5-C). *AtA6K2* gene was inserted into pGBKT7 (BD), and C-terminus 187 amino acid truncated AtABI5 (*AtABI5-N*) and *AtABI5-C* were inserted into pGADT7 (AD). **(D)** AtABI5 interacts with AtS6K2 in bimolecular fluorescence complementation (BiFC) assay. Fluorescence was observed in *Nicotiana tabacum* leaves by agroinfiltration for confocal laser scanning microscopy. The C-terminal part of YFP fused with AtABI5 (AtABI5-cYFP), and the N-terminal part of YFP fused with AtS6K2 (AtS6K2-nYFP). Bars, 50 μm.

To understand how TOR signal modulates ABI5 during seedling growth in *Arabidopsis*, we chose some important genes including *RAPTOR1A*, *RAPTIR1B*, *BRASSINOSTEROID INSENSITIVE 2* (*BIN2*), *S6K1*, and *S6K2* of TOR signaling to identify ABI5 potentially interacting proteins by using the yeast two-hybrid system. We introduced the full length of ABI5 into the Gal4 activation domain of the prey vector pGADT7 (ABI5-AD). The bait and prey vectors were co-transformed into yeast and the protein–protein interaction was identified based on dropout medium lacking Leu, Trp, Ade, and His (Figure 6A). Consistent with previous study, BIN2, a glycogen synthase kinase 3-like kinase, interacted with ABI5 in the yeast two-hybrid system (Hu and Yu, 2014). Additionally, we found that S6K2, a key target protein of TOR kinase, also interacted with ABI5. However, S6K1, a homologous protein of S6K2, did not interact with ABI5 (Figure 6A). To determine which conserved domains of ABI5 interacts with S6K2, we truncated ABI5 to the N-terminus containing C1, C2, and C3 (ABI5-N) and the C-terminus containing bZIP and C4 domains (ABI5-C). Yeast two-hybrid experiments revealed that S6K2 interacted with the full length of ABI5 and the ABI5-C terminus containing bZIP and C4 domains (Figures 6B,C), suggesting the bZIP domain of ABI5 is critical for the ABI5–S6K2 interaction. The ABI5–S6K2 interaction in plant was further verified by BiFC assay. For the BiFC assays, ABI5 was fused to a C-terminal yellow fluorescent protein (YFP) vector (ABI5-cYFP), and the S6K2 protein was fused to an N-terminal YFP vector (S6K2-nYFP). No fluorescence was detected in the negative control experiments. When fused ABI5-cYFP was co-expressed with S6K2-nYFP in leaves of tobacco (*N. benthamiana*), the YFP fluorescence signal was observed in transformed cell nuclei (Figure 6D). These results demonstrated that the ABI5 transcription factor interacts with the S6K2 kinase in plant cell nuclei.

### AtS6K2 Positively Modulates ABA Responses During Seedling Growth

**FIGURE 7:**
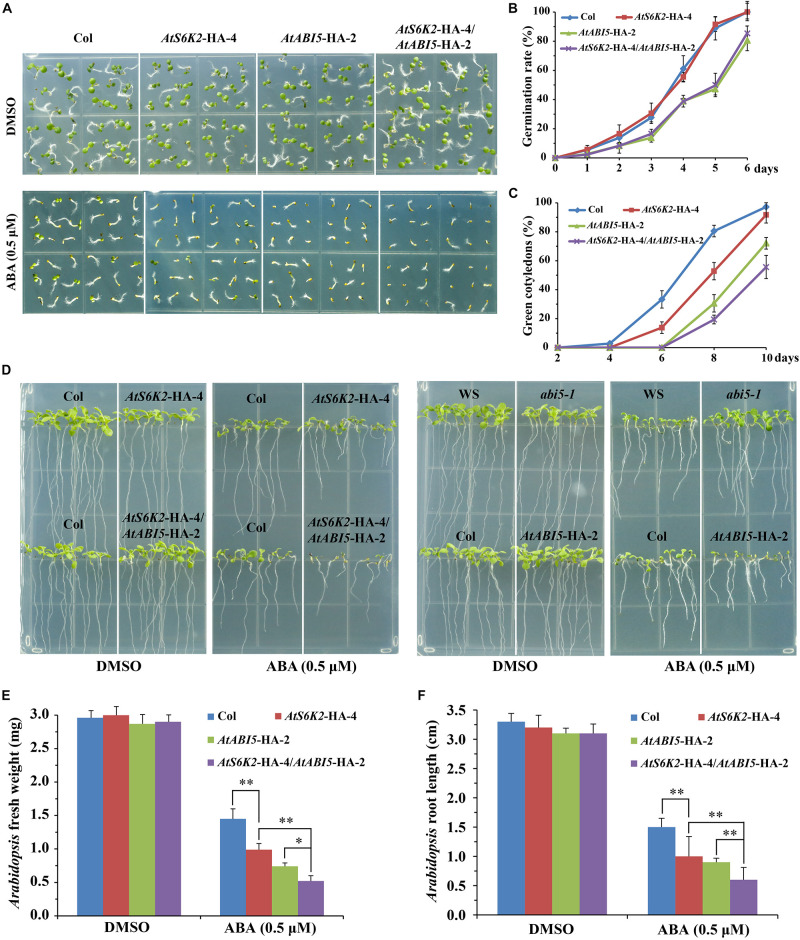
*AtS6K2* overexpression plants intensify the ABA-hypersensitive phenotype of *AtABI5* overexpression plants. **(A)** Phenotypes of indicated seeds germinated on medium containing DMSO and ABA (0.5 μM) for 6 days. Experiments described above were performed three times. Similar results were obtained, and representative results from one experiment were shown. **(B)** Germination rate of indicated seeds. Seed germination was recorded from 0–6 days on medium supplemented with 0.5 μM ABA. Fifty seeds of each genotype were examined for each biological replicate. Error bars indicate means ± SD of three biological replicates. **(C)** Cotyledon greening of indicated seeds. Cotyledon greening was recorded on medium containing 0.5 μM ABA from 2, 4, 6, 8 to 10 days. Fifty seeds of each genotype were examined for each biological replicate. Error bars indicate means ± SD of three biological replicates. **(D)** Phenotypes of indicated seeds sown on plates containing DMSO and ABA (0.5 μM) for 10 days. Experiments described above were performed three times. Similar results were obtained, and representative results from one experiment were shown. **(E,F)** Fresh weight and root length of indicated seeds sown on plates containing DMSO and ABA (0.5 μM) for 10 days. Each graph represents the average of 30 seedlings. Error bars indicate ±SD of three biological experiments. Asterisks denote Student’s *t*-test significant difference (^∗^*P* < 0.05; ^∗∗^*P* < 0.01).

To investigate whether S6K2 protein play a conserved role in modulating ABA signaling in *Arabidopsis*, we used the *P35S*::*AtS6K2*-HA (*AtS6K2*-HA) OE transgenic lines to analyze the role of S6K2 protein in ABA responses. The transcript level of *S6K2* was significantly increased in *AtS6K2*-HA lines (Supplementary Figure 4). We evaluated seed germination and cotyledon greening of the *AtS6K2*-HA-4 line on 1/2 MS medium containing 0.5 μM ABA. Interestingly, *AtS6K2*-HA-4 had no effect on the seed germination, whereas had much lower cotyledon greening than the WT on 1/2 MS containing ABA (Figures 7A–C), which is consistent with the TOR inhibitors-treated BP12-2 phenotype of our previous study. In addition, fresh weight and root length of the *AtS6K2*-HA-4 line was significantly lower than that of the WT in the presence of exogenous ABA during seedling growth (Figures 7D–F). Furthermore, other *AtS6K2*-HA transgenic lines also showed similar ABA response phenotype as the *AtS6K2*-HA-4 line (Supplementary Figure 5). To further confirm the role of S6K2 protein in ABA response, *AtS6K2*-HA-4 line was crossed with *AtABI5*-HA-2 line, and produced the *AtS6K2*-HA-4/*AtABI5*-HA-2 line. As expected, *AtS6K2*-HA-4/*AtABI5*-HA-2 line had much lower cotyledon greening, fresh weight and root length than the *AtS6K2*-HA-4 or *AtABI5*-HA-2 line on 1/2 MS containing 0.5 μM ABA. These results indicated that *AtS6K2*-HA OE plants intensify the ABA-hypersensitive phenotype of *AtABI5*-HA OE plants.

**FIGURE 8:**
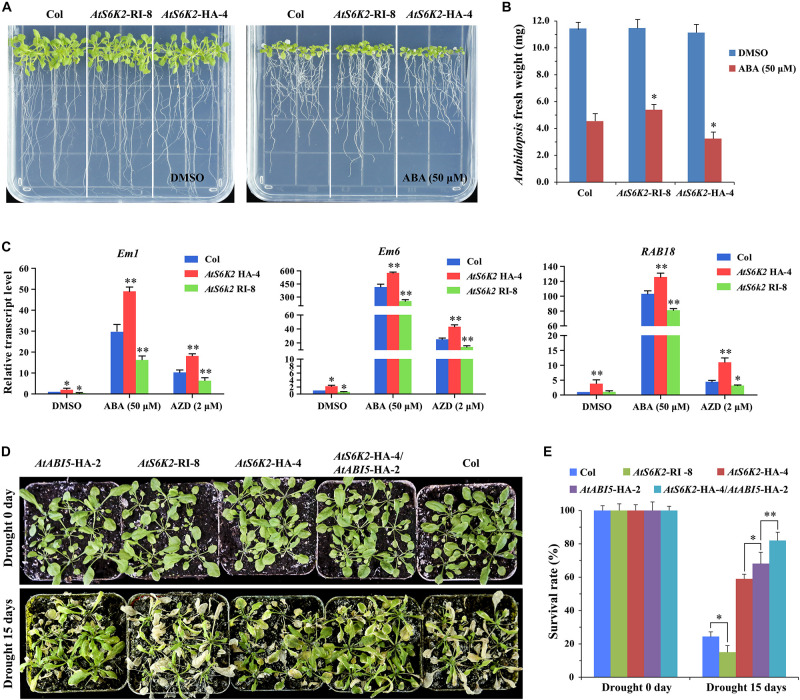
AtS6K2 positively modulates ABA responses during seedling growth in *Arabidopsis*. **(A)** Photographs showing the ABA sensitivity of indicated plants. Seven-day-old seedlings were transferred to 1/2 MS medium with or without 50 μM ABA treated for 10 days. **(B)** Fresh weight of *AtS6K2*-RI, WT and *AtS6K2*-HA *Arabidopsis* plants. Seven-day-old seedlings were transferred to 1/2 MS medium with or without 50 μM ABA treated for 10 days. Each graph represents the average of 10 seedlings. Error bars indicate ±SD of three biological experiments. Asterisks denote Student’s *t*-test significant difference compared with Col (^∗^*P* < 0.05). **(C)** Relative transcript levels of ABA-responsive genes in 7-day-old indicated plants treated with DMSO, ABA (50 μM), and AZD (2 μM) for 48 h. Error bars indicate ±SD of three biological experiments. Asterisks denote Student’s *t*-test significant difference compared with Col (^∗^*P* < 0.05; ^∗∗^*P* < 0.01). **(D)** The transgenic *AtS6K2*-HA *Arabidopsis* plants were resistance to drought. Phenotypes of 30-day-old indicated plant seedlings growing in pots for drought treatment 0 and 15 days. Experiments described above were performed three times. Similar results were obtained, and representative results from one experiment were shown. **(E)** The survival rate of indicated plants growing in pots for drought treatment 0 and 15 days. Three independent biological experiments were carried out to investigate the survival rate of WT and transgenic lines under drought stress. Each graph represents the average of 30 seedlings. Error bars indicate ±SD of three biological experiments. Asterisks denote Student’s *t*-test significant difference (^∗^*P* < 0.05; ^∗∗^*P* < 0.01).

Furthermore, we produced *AtS6K2* RNAi lines (*AtS6K2*-RI) by introducing *P35S*::*AtS6K2*-RI RNAi construct into Col. The transcript level of *S6K2* was significantly decreased in *AtS6K2*-RI lines whereas the transcript level of *S6K1* had no obvious change in *AtS6K2*-HA or *AtS6K2*-RI lines compared with WT plants (Supplementary Figure 4). *AtS6K2*-RI lines were insensitive to ABA compared with Col whereas *AtS6K2*-HA lines were hypersensitive to ABA on 1/2 MS medium containing 50 μM ABA (Figures 8A,B and Supplementary Figure 6). Additionally, we also analyzed the expression patterns of ABA-responsive marker genes in the *AtS6K2*-RI and *AtS6K2*-HA transgenic lines. The results showed that the expression of ABA-responsive genes including *Em1*, *Em6*, and *RAB18* was significantly upregulated in the *AtS6K2*-HA-4 seedlings with ABA and AZD treatment. By contrast, the expression level of these marker genes was reduced in the *AtS6K2*-RI-8 transgenic line compared with the Col (Figure 8C). Among these marker genes, the expression level of *Em6* showing a largest difference in ABA and AZD treated seedlings. Because *Em1* and *Em6* are known ABI5 target genes (Carles et al., 2002), these results suggested that S6K2 induced expression of ABI5-regulated genes in *Arabidopsis*.

To determine the effect of S6K2 on seedling growth in environmental stress, we examined the phenotypes of the *AtS6K2*-HA OE transgenic line and *AtS6K2* RNAi line following drought treatment. *AtABI5*-HA-2 and *AtS6K2*-HA-4 lines showed enhanced resistance to drought treatment while *AtS6K2*-RI-8 line was sensitive to drought. The survival rate of *AtABI5*-HA-2 and *AtS6K2*-HA-4 transgenic lines were increased compared with Col under drought treatment, while that of *AtS6K2*-RI-8 line was strongly reduced (Figures 8D,E). Moreover, *AtS6K2*-HA-4/*AtABI5*-HA-2 line showed more resistance to drought and more survival rate than *AtABI5*-HA-2 or *AtS6K2*-HA-4 line. Together, these results demonstrated that AtS6K2 positively modulates ABA responses during seedling growth in *Arabidopsis*.

## Discussion

Phytohormone ABA plays key roles in vegetative, seed dormant and germination, and biotic and abiotic stresses such as drought, salt stress, and pathogen infection (Jurriaan et al., 2009; Zhao et al., 2016; Vishwakarma et al., 2017; Kumar et al., 2019). ABI5 is a key transcription factor in regulating the expression of ABA-responsive genes in the ABA signaling pathway (Skubacz et al., 2016; Zhu et al., 2020). Previous study revealed that ABI5 was phosphorylated at Thr-35, Ser-36, Ser-41, Ser-42, Ser-138, Ser-139, Ser-145, and Thr-201 residues in *Arabidopsis* (Hu and Yu, 2014; Yu et al., 2015), of which Ser-42, Ser-145, and Thr-201 sites of ABI5 were phosphorylated by SnRK2s protein kinases (Fujii and Zhu, 2009). Among those phospho-amino sites, Ser-41, Thr-35, Ser-36, Ser-368, and Ser-372 on ABI5 were BIN2 phosphorylation sites. BIN2, an important negative regulator of brassinosteroid signal, interacts with ABI5 to mediate the antagonism of BR and ABA during seed germination in *Arabidopsis* (Hu and Yu, 2014). Additionally, calcineurin B-like interacting protein kinase 26 (CIPK26) and CIPK11 also phosphorylate ABI5 to regulate ABA inhibition of seed germination in *Arabidopsis* (Lyzenga et al., 2013). In this study, we found that S6K2 interacted with ABI5 to respond environmental stresses. Interestingly, the S6K1 of S6K2 homolog did not interact with ABI5. S6Ks are key target proteins of TOR signal, and TOR can directly phosphorylate S6Ks to control plant growth and development (Schepetilnikov et al., 2013; Xiong et al., 2017). Recent studies have shown that S6K2 can phosphorylate the BR signaling pathway negative regulator BIN2 to regulate the process of plant from heterotrophic to photoautotrophic. Although the protein sequence of S6K1 is highly similar to S6K2, S6K1 does not phosphorylate BIN2 in *Arabidopsis* (Xiong et al., 2017). Furthermore, AtS6K1 is only localized in the cytoplasm, but AtS6K2 is mainly localized in the nucleus and a small part in the cytoplasm (Mahfouz et al., 2006), indicating that S6K1 and S6K2 have different molecular functions to regulate different biological processes. Interestingly, ABI5 is predicted to be a nucleocytoplasmic protein, which contains two basic NLSs and a leucine-rich NES (Liu and Stone, 2013). Recent studies showed that ABI5 was mainly located in the nucleus whereas ABI5 was degraded in both the nucleus and cytoplasm (Lopez-Molina et al., 2003; Liu and Stone, 2013), indicating that ABI5 is also present in the cytoplasm. Collectively, these results indicated that ABI5 and S6K2 have overlapping subcellular localization in *Arabidopsis*, implying the function of ABI5 and S6K2 in ABA signaling.

We further explored the function of AtS6K2 in ABA responses. *AtS6K2* OE lines had no effect on the seed germination whereas arrested cotyledon greening of postgerminative and seedling growth under stress conditions. Furthermore, *AtS6K2* OE lines enhanced resistance to drought treatment while *AtS6K2* interference lines were sensitive to drought. These results indicated that AtS6K2 positively regulated ABA responses during seedling growth in *Arabidopsis*. Recent study showed that the viral genome-linked protein (VPg) of *Turnip mosaic virus* and *Potato virus A* interacted with AtS6K2 in plant cytoplasm and nucleus (Rajamaki et al., 2017). The VPg–AtS6K2 interaction has the potential to interfere with AtS6K2 functions, implying that AtS6K2 has functions in fighting against biotic stresses. Collectively, these observations suggest that AtS6K2 plays an important role in biotic and abiotic stresses.

In plants, TOR is an important regulator of the switch between the plant growth and stress response. TOR controls translational initiation/reinitiation by phosphorylating the S6K1 and translation initiation factor elF3h (Schepetilnikov et al., 2013). The phytohormone auxin promotes activation of TOR through a physical interaction between TOR and GTPase ROP2 to promote the translation reinitiation of uORF-containing mRNAs and activation of root meristem (Li et al., 2017; Schepetilnikov et al., 2017). Sugar-TOR signaling stabilizes the BR signaling key transcription factor BZR1 to promote carbon availability and plant growth (Xiong et al., 2013; Zhang et al., 2016; Lee and Seo, 2017). Besides, TOR and ABA signals have a conserved phospho-regulatory feedback mechanism to balance plant growth and stress response (Wang et al., 2018). In this study, we found that TOR inhibitors RAP and AZD enhanced the expression of ABA biosynthesis and ABA-responsive genes. Inhibition of TOR increased ABA content, which activated ABA-responsive genes to respond stresses, and in turn resulting in plant growth arrest. Surprisingly, RAP and AZD had no effect on seed germination. Instead, RAP and AZD enhanced the inhibitory effect of ABA on cotyledon greening and seedling growth during postgerminative growth. Similar results were also found in cytokinin mediated seed germination and cotyledon greening of postgerminative growth (Guan et al., 2014). Previous studies have shown that rapamycin and asTORis synergistically inhibited TOR activity and plant growth (Xiong et al., 2017). Besides, methyl jasmonate and TOR inhibitors also synergistically inhibit plant growth in *Arabidopsis* (Song et al., 2017). We found that TOR inhibitors and ABA had synergy effects based on the Fa-CI curve, implying an interaction between TOR and ABA signals. Furthermore, ABA signal deficient mutants screening indicated that *abi5-1* was insensitive to AZD whereas *ABI5* OE lines were hypersensitive to AZD. Our results further suggested that TOR-inhibition induced excessive accumulation of ABI5 protein in *Arabidopsis*. Y2H and BiFC assays showed that ABI5 interacts with S6K2 *in vivo*, and *S6K2* OE lines showed enhanced resistance to drought treatment while *S6K2* RNAi lines were sensitive to drought in *Arabidopsis*. Collectively, these findings suggested that ABI5 plays an important role in mediating the crosstalk between TOR and ABA signals. Importantly, ABI5 interacts with S6K2 to positively modulate ABA responses during seedling growth in *Arabidopsis*.

## Data Availability Statement

The original contributions presented in the study are included in the article/Supplementary Material, further inquiries can be directed to the corresponding author/s.

## Author Contributions

MR, LL, and TZ designed the experiments and wrote the manuscript. LL, TZ, YS, and EF performed the experiments. LL and LF analyzed the data. All authors contributed to the article and approved the submitted version.

## Conflict of Interest

The authors declare that the research was conducted in the absence of any commercial or financial relationships that could be construed as a potential conflict of interest.

## Supplementary Material

Supplementary Figure 1 TOR kinase inhibitors RAP and AZD have no effect on seed germination. **(A)** Phenotypes of BP12-2 and WT seeds sown on plates containing DMSO, ABA (0.5 μM), RAP (0.35 μM), and AZD (0.5 μM) for 3 days. **(B)** Germination rate of BP12-2 and WT seeds sown on plates containing DMSO, ABA (0.5 μM), RAP (0.35 μM), and AZD (0.5 μM) for 0, 1, 2, and 3 days. Fifty seeds of each treatment were examined for each biological replicate. Error bars indicate means ± SD of three biological replicates.

Supplementary Figure 2 *abi5-1* was insensitive to AZD whereas *ABI5* overexpression lines were hypersensitive to AZD. **(A)** Phenotypes of *abi5-1*, *ABI5* overexpression lines (OE) and WT seeds sown on plates containing DMSO, ABA (1 μM), and AZD (1 μM) for 7 days. **(B)** Phenotypes of *abi5-1*, *ABI5* overexpression lines (OE) and WT seeds sown on plates containing DMSO, ABA (1 μM), and AZD (1 μM) for 14 days.

Supplementary Figure 3 GUS staining of *ABI5-GUS* OE12 transgenic line. **(A)** GUS staining of leaves of 7-day-old *ABI5-GUS* OE12 plants treated with DMSO, ABA (50 μM), and AZD (2 μM) for 48 h. **(B)** GUS staining of roots of 7-day-old *ABI5-GUS* OE12 plants treated with DMSO, ABA (50 μM), and AZD (2 μM) for 48 h. **(C)** GUS activity of the seedlings of *ABI5-GUS* OE12 plants, plants were treated as described in **(A)**. Asterisks denote Student’s *t*-test significant difference compared with DMSO (^∗∗^*P* < 0.01).

Supplementary Figure 4 The expression levels of *S6K2* and *S6K1* in *AtS6K2*-HA lines and *AtS6K2*-RI lines. Error bars indicate ±SD of three biological experiments.

Supplementary Figure 5 *AtS6K2* overexpression lines were sensitive to ABA. **(A)** Phenotypes of indicated seeds sown on plates containing DMSO and ABA (0.5 μM) for 10 days. Experiments described above were performed three times. Similar results were obtained, and representative results from one experiment were shown. **(B)** Fresh weight and root length of indicated seeds sown on plates containing DMSO and ABA (0.5 μM) for 10 days. Each graph represents the average of 30 seedlings. Error bars indicate ±SD of three biological experiments. Asterisks denote Student’s *t*-test significant difference compared with Col (^∗^*P* < 0.05; ^∗∗^*P* < 0.01).

Supplementary Figure 6 *AtS6K2*-RI lines were insensitive to ABA whereas *AtS6K2*-HA lines were hypersensitive to ABA compared with Col. **(A)** Photographs showing the ABA sensitivity of indicated plants. Seven-day-old seedlings were transferred to 1/2 MS medium with or without 50 μM ABA treated for 10 days. **(B)** Fresh weight and root length of indicated seedlings. Seven-day-old seedlings were transferred to 1/2 MS medium with or without 50 μM ABA treated for 10 days. Each graph represents the average of 10 seedlings. Error bars indicate ±SD of three biological experiments. Asterisks denote Student’s *t*-test significant difference compared with Col (^∗^*P* < 0.05).
