# Supplementary figures and images for: ABSCISIC ACID INSENSITIVE5 Interacts With RIBOSOMAL S6 KINASE2 to Mediate ABA Responses During Seedling Growth in Arabidopsis
Source: Front Plant Sci. 2021 Jan 18;11:598654. doi: 10.3389/fpls.2020.598654 (PMC7847994; doi:10.3389/fpls.2020.598654)

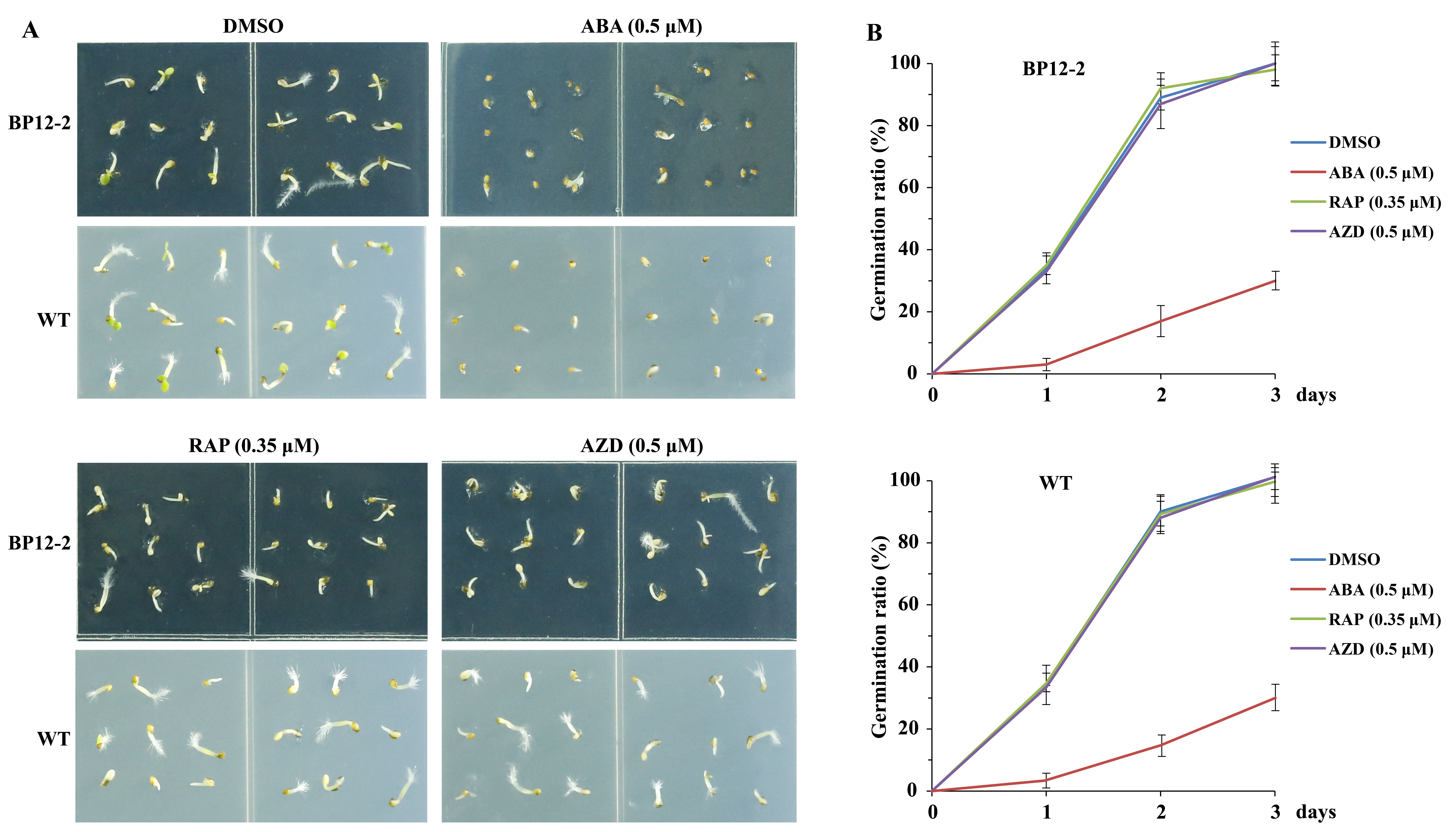

Supplement: Supplementary Figure 1 — TOR kinase inhibitors RAP and AZD have no effect on seed germination. (A) Phenotypes of BP12-2 and WT seeds sown on plates containing DMSO, ABA (0.5 μM), RAP (0.35 μM), and AZD (0.5 μM) for 3 days. (B) Germination rate of BP12-2 and WT seeds sown on plates containing DMSO, ABA (0.5 μM), RAP (0.35 μM), and AZD (0.5 μM) for 0, 1, 2, and 3 days. Fifty seeds of each treatment were examined for each biological replicate. Error bars indicate means ± SD of three biological replicates. [file Image_1.JPEG]

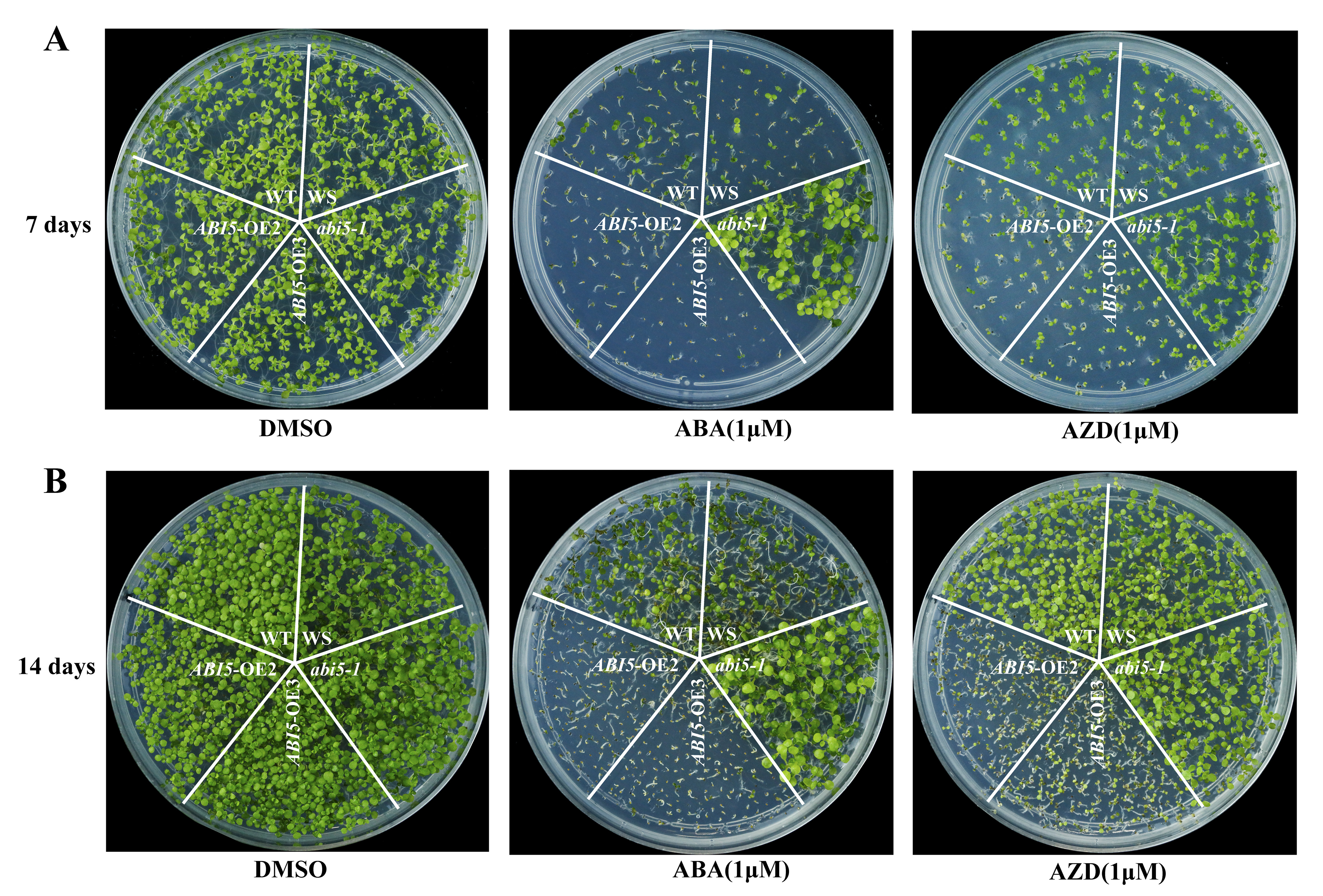

Supplement: Supplementary Figure 2 — abi5-1 was insensitive to AZD whereas ABI5 overexpression lines were hypersensitive to AZD. (A) Phenotypes of abi5-1, ABI5 overexpression lines (OE) and WT seeds sown on plates containing DMSO, ABA (1 μM), and AZD (1 μM) for 7 days. (B) Phenotypes of abi5-1, ABI5 overexpression lines (OE) and WT seeds sown on plates containing DMSO, ABA (1 μM), and AZD (1 μM) for 14 days. [file Image_2.JPEG]

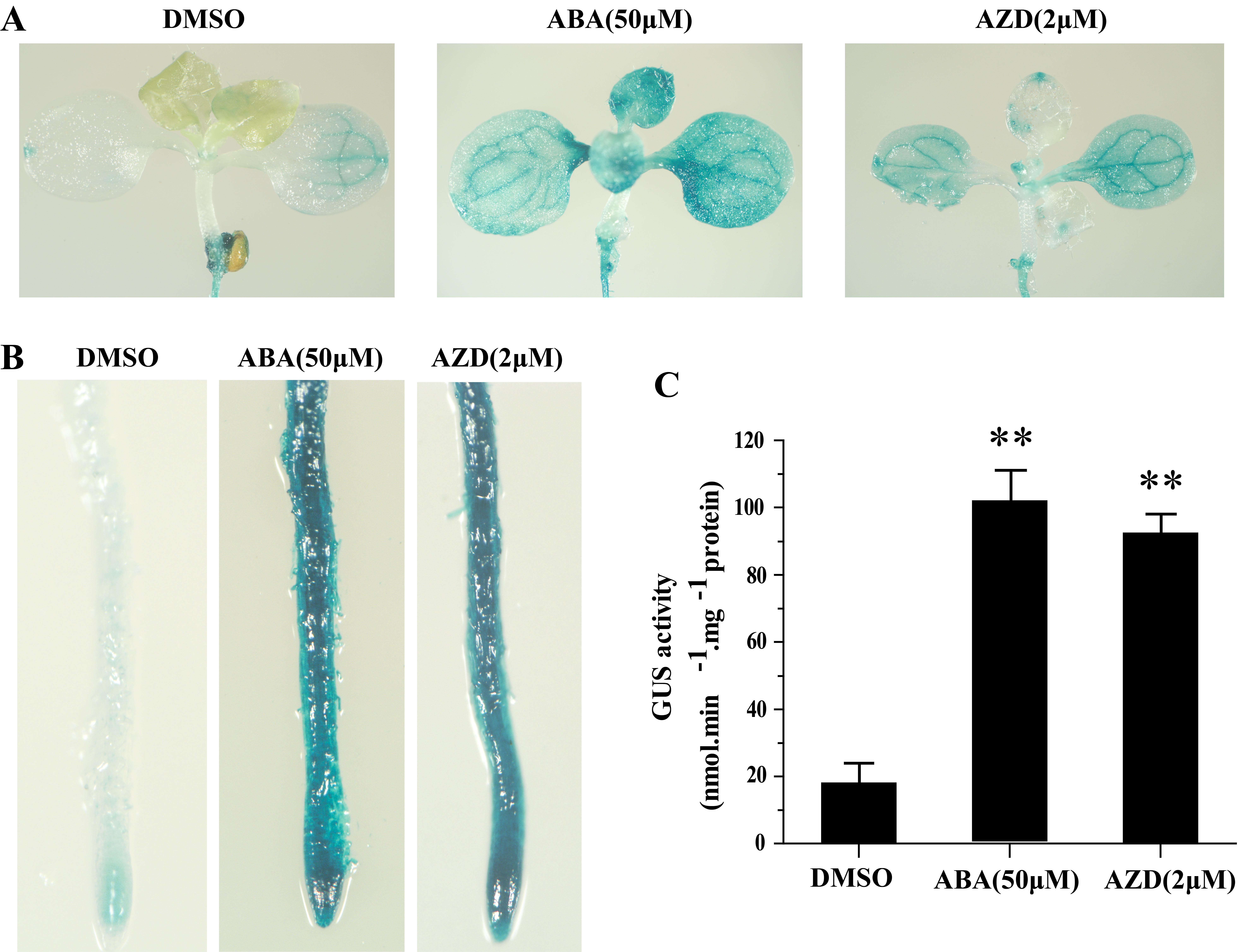

Supplement: Supplementary Figure 3 — GUS staining of ABI5-GUS OE12 transgenic line. (A) GUS staining of leaves of 7-day-old ABI5-GUS OE12 plants treated with DMSO, ABA (50 μM), and AZD (2 μM) for 48 h. (B) GUS staining of roots of 7-day-old ABI5-GUS OE12 plants treated with DMSO, ABA (50 μM), and AZD (2 μM) for 48 h. (C) GUS activity of the seedlings of ABI5-GUS OE12 plants, plants were treated as described in (A). Asterisks denote Student’s t-test significant difference compared with DMSO (∗∗P < 0.01). [file Image_3.JPEG]

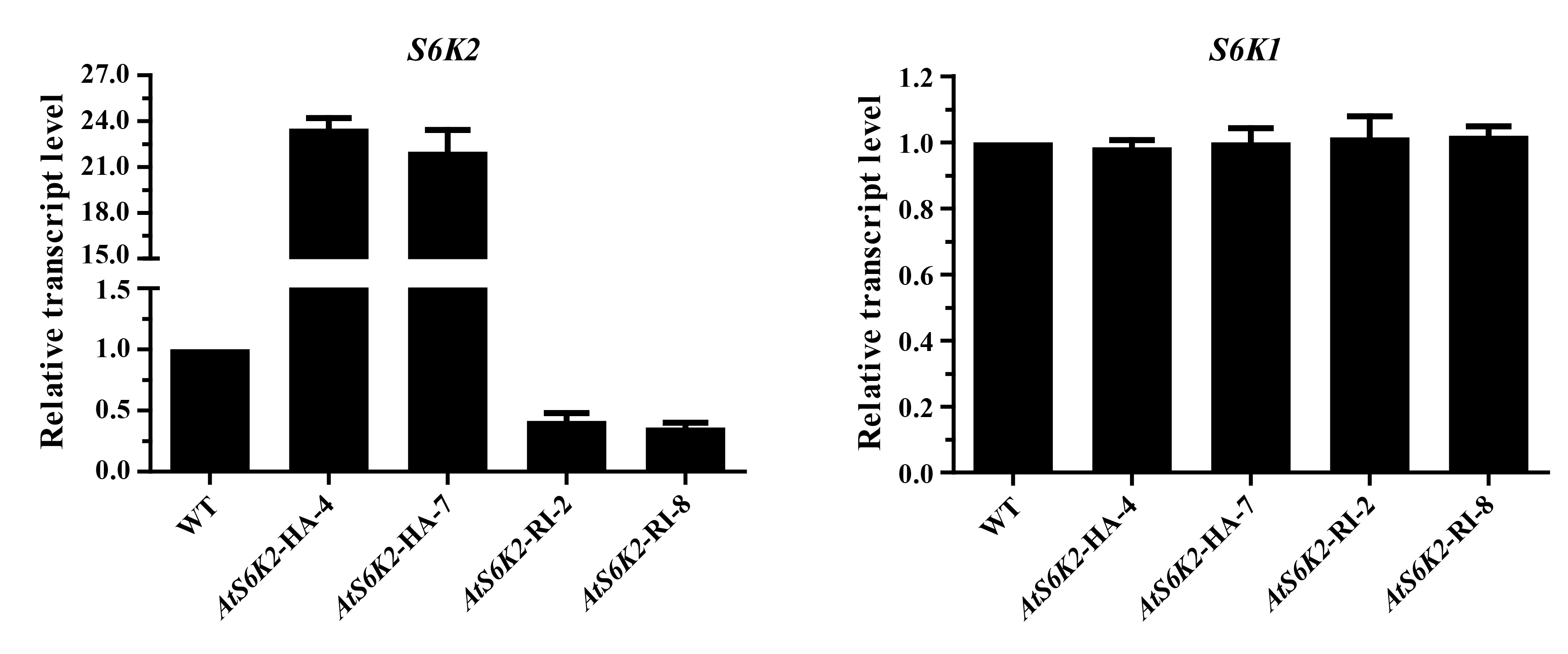

Supplement: Supplementary Figure 4 — The expression levels of S6K2 and S6K1 in AtS6K2-HA lines and AtS6K2-RI lines. Error bars indicate ±SD of three biological experiments. [file Image_4.JPEG]

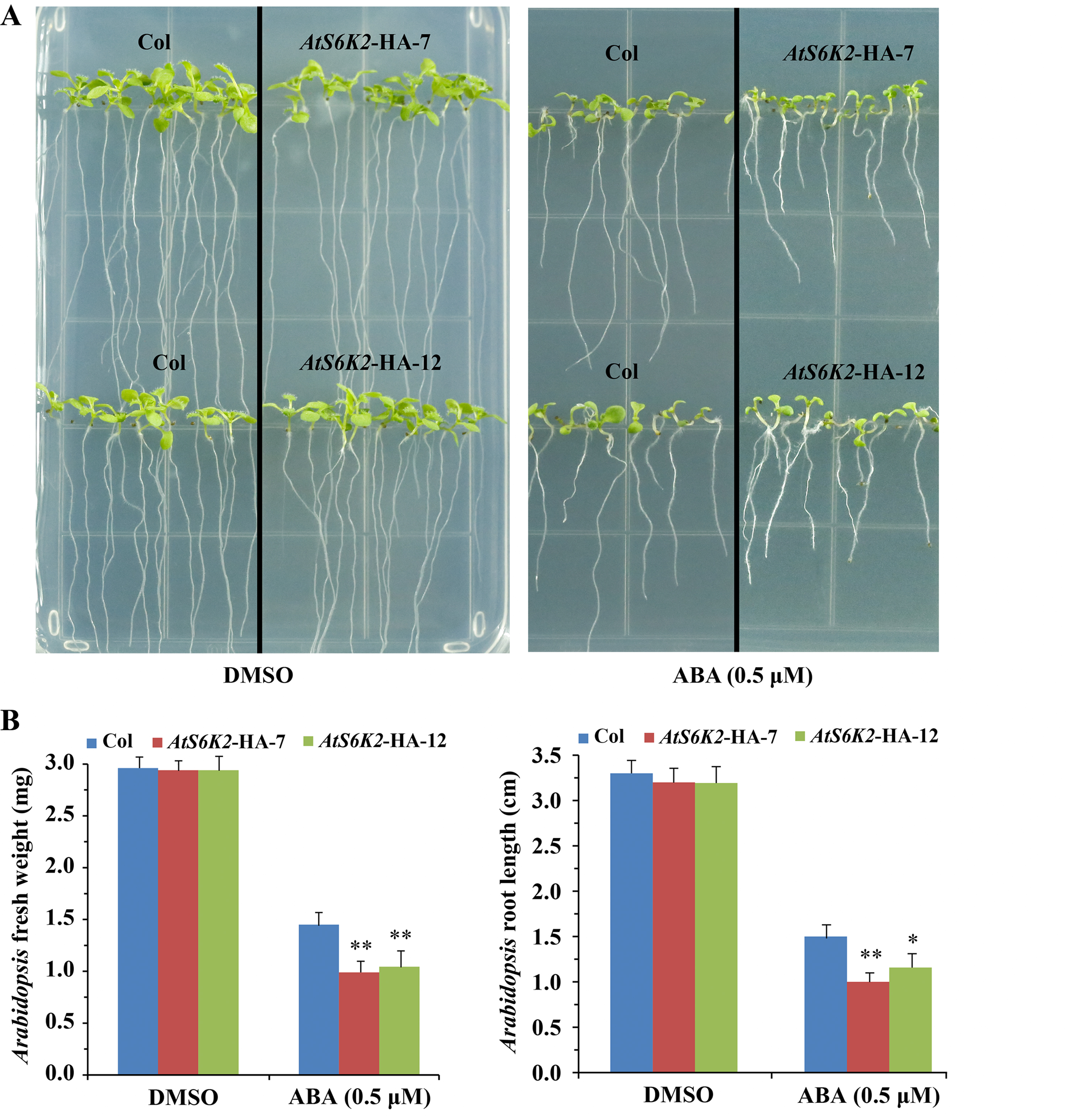

Supplement: Supplementary Figure 5 — AtS6K2 overexpression lines were sensitive to ABA. (A) Phenotypes of indicated seeds sown on plates containing DMSO and ABA (0.5 μM) for 10 days. Experiments described above were performed three times. Similar results were obtained, and representative results from one experiment were shown. (B) Fresh weight and root length of indicated seeds sown on plates containing DMSO and ABA (0.5 μM) for 10 days. Each graph represents the average of 30 seedlings. Error bars indicate ±SD of three biological experiments. Asterisks denote Student’s t-test significant difference compared with Col (∗P < 0.05; ∗∗P < 0.01). [file Image_5.JPEG]

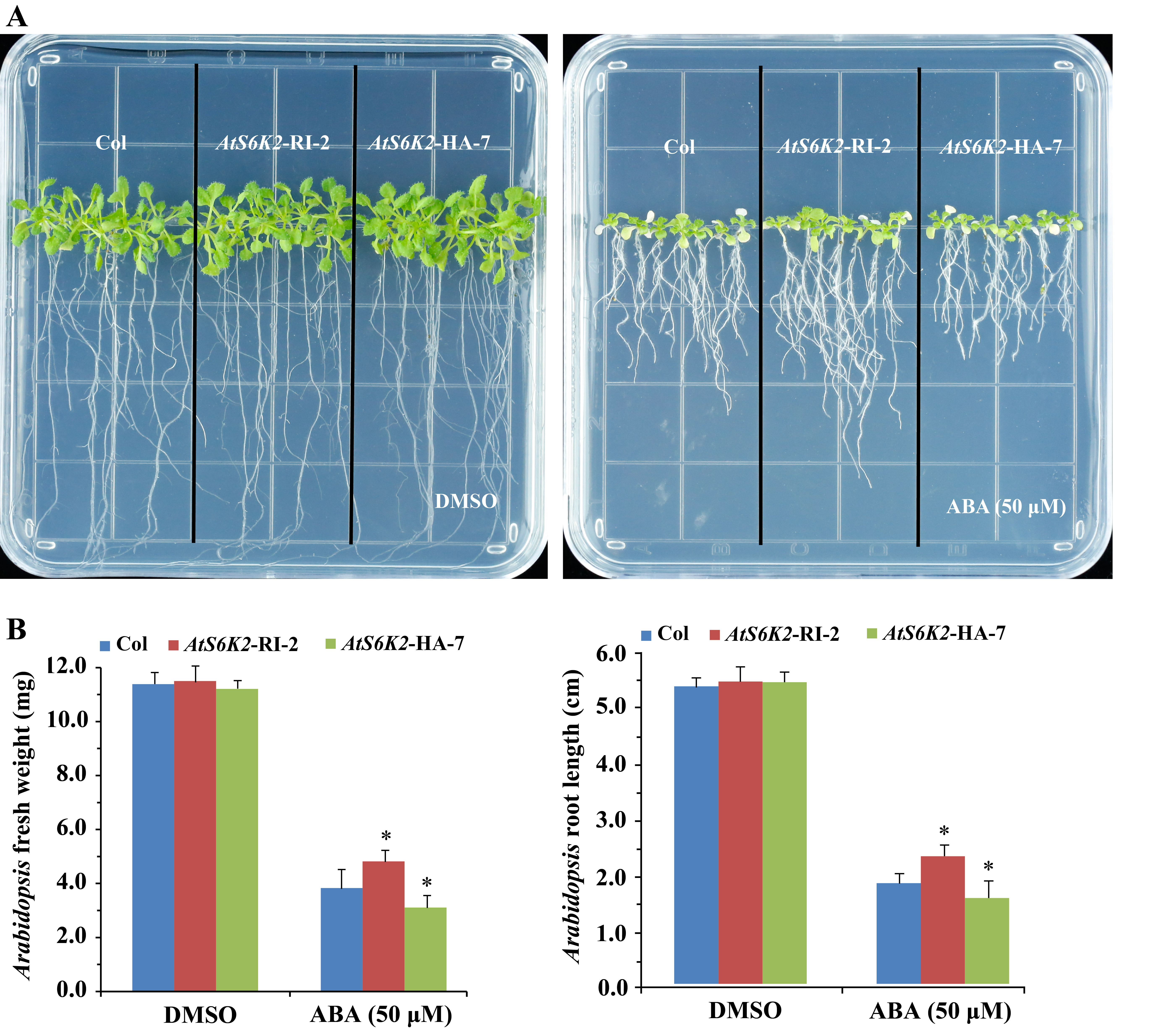

Supplement: Supplementary Figure 6 — AtS6K2-RI lines were insensitive to ABA whereas AtS6K2-HA lines were hypersensitive to ABA compared with Col. (A) Photographs showing the ABA sensitivity of indicated plants. Seven-day-old seedlings were transferred to 1/2 MS medium with or without 50 μM ABA treated for 10 days. (B) Fresh weight and root length of indicated seedlings. Seven-day-old seedlings were transferred to 1/2 MS medium with or without 50 μM ABA treated for 10 days. Each graph represents the average of 10 seedlings. Error bars indicate ±SD of three biological experiments. Asterisks denote Student’s t-test significant difference compared with Col (∗P < 0.05). [file Image_6.JPEG]
