# Supplementary material for: ABSCISIC ACID INSENSITIVE5 Interacts With RIBOSOMAL S6 KINASE2 to Mediate ABA Responses During Seedling Growth in Arabidopsis
Source: Front Plant Sci. 2021 Jan 18;11:598654. doi: 10.3389/fpls.2020.598654 (PMC7847994; doi:10.3389/fpls.2020.598654)
Supplement: Supplementary file 7 [file Table_1.DOCX]

Supplementary Material

**Supplementary Tables**

**Supplementary Table 1.** Primers for quantitative real-time PCR in this study

| Primers | Sequences (5’-3’) | Length |
| --- | --- | --- |
| *AtNCED3* F | 5’-AAAGCCATCGGTGAGCTTCA-3’ | 20 |
| *AtNCED3* R | 5’-GCAGCTCTGGCGTAGAATAGC-3’ | 21 |
| *AtAAO4* F | 5’-AAGACAAACCTTCCGAGCAG-3’ | 20 |
| *AtAAO4* R | 5’-TCACAACATCCACGTCCATC-3’ | 20 |
| *AtABA3* F | 5’-GCTGTTAGGGTTTCGTTTGG-3’ | 20 |
| *AtABA3* R  *AtACTIN2* F  *AtACTIN2* R  *AtZEP* F  *AtZEP* R  *AtRD29A* F  *AtRD29A* R  *AtRD29B* F  *AtRD29B* R  *AtRD22* F  *AtRD22* R  *AtRAB18* F  *AtRAB18* R  *AtEm6* F  *AtEm6* R  *AtEm1* F  *AtEm1* R  *AtS6K2* F  *AtS6K2* R  *AtS6K1* F  *AtS6K1* R | 5’-CCTTCCACTGACGACGGTTC-3’  5’-GCACTTGCACCAAGCAGCAT-3’  5’-CCTTTCAGGTGGTGCAACGAC-3’  5’-ATCCCTTCGTCTCAGGTTTC-3’  5’-TCACATAGGTTCCGTGTTCG-3’  5’-GGACAAAGGTGTTTCCTGTCG-3’  5’-TCCGATGTAAACGTCGTCC-3’  5’-GCAAGCAGAAGAACCAATC-3’  5’-CTTTGGATGCTCCCTTCTC-3’  5’-TTCGCGGTGTTCTACTGCC-3’  5’-CGGAACCGCGTAGACGG-3’  5’-GGGACTGAAGGCTTTGGAAC-3’  5’-GATGACCTGGCAACTTCTCC-3’  5’-AACAAGAGAAGAAGCAGCTGGAT-3’  5’-TCTTGGTCCTGAATTTGGATTCGT-3’  5’-ATGGCGTCAAAGCAACTGAGC-3’  5’-TCACTTGTTGGTGAACTTTGACTC-3’  5’-TCGTCTCCTTCCTCAAGCAT-3’  5’-TGGAGGGCTGATGGATAAAG-3’  5’-TCTGCAGCATCCACTGTTTC-3’  5’-GAATTTGAAGGGCTGATGGA-3’ | 20  20  21  20  20  21  19  19  19  19  19  20  20  23  24  21  24  20  20  20  20 |

**Supplementary Table 2.** The GI50 values of each drug used in BP12-2 line

| Drug | GI50 value |
| --- | --- |
| RAP | 0.35 μM |
| AZD | 0.5 μM |
| ABA | 0.5 μM |
| ABA+RAP | 0.1 μM+0.05 μM |
| ABA+AZD | 0.1 μM+0.1 μM |
